# Editorial: Motor Control of Gait and the Underlying Neural Network in Pediatric Neurology

**DOI:** 10.3389/fnhum.2019.00226

**Published:** 2019-07-02

**Authors:** Pieter Meyns, Maud van den Bogaart, Kyra Theunissen, Marjolein M. van der Krogt, Els Ortibus, Kaat Desloovere

**Affiliations:** ^1^Rehabilitation Research (REVAL), Faculty of Rehabilitation Sciences, Hasselt University, Diepenbeek, Belgium; ^2^Department of Human Movement Sciences, Vrije Universiteit Amsterdam, Amsterdam Movement Sciences, Amsterdam, Netherlands; ^3^Department of Human Biology and Movement Sciences, NUTRIM School of Nutrition and Translational Research in Metabolism, Maastricht University, Maastricht, Netherlands; ^4^Department of Rehabilitation Medicine, Vrije Universiteit Amsterdam, Amsterdam Movement Sciences, Amsterdam UMC, Amsterdam, Netherlands; ^5^Centre for Developmental Disabilities, University Hospital Leuven, Leuven, Belgium; ^6^Department of Rehabilitation Sciences, Faculty of Kinesiology and Rehabilitation Sciences, KU Leuven, Leuven, Belgium; ^7^Clinical Motion Analysis Laboratory, CERM, University Hospital Leuven, Leuven, Belgium

**Keywords:** gait, cerebral palsy, pediatric neurology, MRI, upper limb, ataxia, tDCS, Duchenne muscular dystrophy

## Introduction

Researchers around the world strive toward better understanding of the complexity and causes of movement disorders due to nervous system disease. Such research has mainly focused on adult neurological disorders such as stroke and Parkinson's disease, while significantly fewer studies have investigated pediatric populations. This may be caused by the complexity of nervous system diseases in children due to the combination of changes caused by disease and development. Therefore, within this research topic, we aimed at stimulating researchers to address important aspects of motor control and the underlying neural network in pediatric neurology.

Thirteen papers have been bundled in this eBook, including 1 review paper and 12 original research papers. Most of these papers focused on cerebral palsy (CP), which is not unexpected as CP is the most common developmental disorder associated with lifelong movement and posture disability (Aisen et al., 2011). One paper focused on Duchenne muscular dystrophy (DMD) and one on early-onset ataxia (EOA). We aimed to bundle papers on neural causes of movements disorders and their relation with motor function. Two included studies examined the link between brain lesions and motor control in CP (Grecco et al.,; Mailleux et al.,) and one review focused on the neural correlates of gait abnormalities in CP (Zhou et al.,). Understanding the neural control of movements in children with neurological disorders can help to improve existing or develop new rehabilitation strategies. Therefore, we also included papers that investigated (factors that influence) the control of movements, e.g., weakness, instability, and spasticity, which impact motor independence. None, however, focused on neuroimaging during gait.

## Contributions to the Research Topic

Two papers focused on upper limb reaching and grasping in CP. Mailleux et al. found a relation between structural brain damage and upper limb kinematics in unilateral CP. They found more aberrant patterns in children with cortical and deep gray matter lesions compared to children with periventricular white matter lesions. Additionally, lesion location and extent had a greater influence on the kinematics in children with cortical and deep gray matter lesions. Importantly, damage to the posterior limb of the internal capsule was a predictor for the movement pattern. Simon-Martinez et al. estimated the impact of motor impairments on upper body kinematics. The results indicated that children with unilateral CP showed altered movement patterns in all joints during a large percentage of the movement cycle compared to typically developing (TD) children. Spasticity and muscle weakness had a negative effect on the movement pattern.

One contribution focused on gait in EOA. Lawerman et al. investigated the construct validity of the SARA_GAIT/POSTURE_ sub-scales (Scale for Assessment and Rating of Ataxia) by relating these to existing balance scales (e.g., pediatric balance scale) and to a functional mobility classification system (GMFCS). They found a high construct validity for the SARA_GAIT/POSTURE_ sub-scales. However, the scores discriminated insufficiently between the influence of ataxia and muscle weakness, which suggests the possible influence of ataxia (and muscle weakness) on the control of gait and posture in EOA.

Several contributions have identified important factors that influence gait in children with CP. Using muscle synergy analysis, Goudriaan et al. compared total variance accounted for by one synergy during gait in CP, which have neural and non-neural muscle weakness, with children with DMD, who have non-neural muscle weakness. They found that the complexity of the control of gait was not influenced by the non-neural constraints of muscle weakness, as the total variance accounted for by one synergy was similar between both groups. Contrarily, the importance of the influence of neural muscle weakness on the control of gait in CP was highlighted by a clear association between plantar flexor weakness and reduced synergy complexity in CP. This is in agreement with a paper by Nieuwenhuys et al. who examined the construct validity of a new gait classification system for CP. They were able to confirm that most joint patterns during gait are characterized by different patient-specific characteristics (such as age and GMFCS-level) and that they are often associated with muscle weakness and spasticity.

To reduce the negative effect of spasticity on gait, Botulinum Toxin treatment (BTX) may be an option. BTX, however, has been suggested to induce muscle weakness which may deteriorate gait in CP. Eek and Himmelmann found that voluntary plantar flexor muscle strength did not decrease after BTX-injections, but seemed to increase at 6-months follow-up. The muscle strength improvement and reduction of spasticity coincided with small improvements in gait kinematics.

A person's body weight may also influence gait in CP. To assess the effectiveness of interventions on gait in CP [as in (Eek and Himmelmann)], pre-post three-dimensional gait analyses are performed, usually with a considerable amount of time between measurements. Meyns et al. however, showed that clinical gait analysts should consider the negative effect of increased weight between pre-post measurements to avoid misinterpretation of interventions. They found that adding 10% body weight in children with CP, had detrimental effects on their spatio-temporal parameters, kinematics, and kinetics. As such, rehabilitation in CP should counteract overweight and obesity.

Also arm movements may have an effect on gait, as previous observational research indicated that children with CP show altered arm movements during walking related to instability (Meyns et al., 2011, 2012, 2016a). In the study by Delabastita et al. children with CP and TD children were required to walk with or without their arms free to swing while assessing trunk movements and gait stability. The results indicated that gait instability and trunk movements increased when arm swing was restricted in children with bilateral CP compared to TD children and children with unilateral CP. A related article by Meyns et al. focused on the possible contribution of the arm movements during gait on propulsion of the center of mass (CoM) in CP. They found that, even though it appeared that the contribution of the arm movements to propulsion of the CoM was larger in CP than in TD children, the contribution of the arms to forward propulsion was negligible compared to that of the legs and gravity. As such, the arms seem to have an important role in stability, but not in propulsion.

One contribution to the topic investigated neural correlates of gait in CP. Grecco et al. investigated the responsiveness to transcranial direct current stimulation combined with gait training. Their results revealed that the presence of a motor evoked potential in the quadriceps muscle was a predictor for gait velocity, while the presence of deeper (subcortical) injuries of the internal capsule was a predictor of gait kinematics and gross motor function.

Two contributions addressed motor learning in CP. Damiano et al. found that children with unilateral CP did not demonstrate poorer learning or retention of their gait pattern in a unilateral perturbation treadmill paradigm compared to TD children. In a follow-up paper by Bulea et al. they used a dynamical systems approach to provide insights in the complexity of the neural control of the legs in unilateral CP. The authors suggested that each leg and each type of walking has distinct neural circuits which can be adapted independently. As such, gait symmetry can be improved in the short term. This highlights the possible use of such paradigms in gait rehabilitation to increase step symmetry in unilateral CP.

Finally, Zhou et al. provided a comprehensive review on brain injuries in CP, associated neuromuscular deficits and gait abnormalities and their neural correlates with a focus on implications for rehabilitation. One of the findings was that only few studies (Meyns et al., 2016b) have examined the neural correlates of motor deficits and gait abnormalities in CP. Nevertheless, to truly assess the underlying neural networks of gait in pediatric neurology, future studies should implement neuroimaging techniques during gait (Makeig et al., 2009; Gwin et al., 2010; Wagner et al., 2012) in these populations which will provide good evidence for the motor control of gait.

With the current research topic, we stimulated researchers to focus on movement control and the underlying neural network in pediatric neurology. It has been shown that there are apparent changes in the relation between brain functional connectivity and the progression of walking and gross motor abilities from infancy to the toddler period (Marrus et al., 2018). Future research may focus on differences in the alterations of these network-level brain–behavior relationships between natural development or neurodevelopmental conditions. Given the scarcity of research on the neural correlates and the underlying neural networks of gait in pediatric neurology, we urge researchers and funding agencies to invest time and resources on this topic as this will help to discover targeted treatment opportunities that can substantially improve functional outcomes for these children.

## Author Contributions

PM was topic editor of the Special Research Topic. MvdK, EO, and KD were co-editors of the Special Research Topic. PM wrote the paper. MvdB, KT, MvdK, EO, and KD edited and revised the paper.

### Conflict of Interest Statement

The authors declare that the research was conducted in the absence of any commercial or financial relationships that could be construed as a potential conflict of interest.
